# Qualitative differences in perspective on children’s quality of life between children with cerebral palsy and their parents

**DOI:** 10.1186/s41687-023-00656-x

**Published:** 2023-11-20

**Authors:** Elena Swift, Lisa Gibbs, Dinah Reddihough, Andrew Mackinnon, Elise Davis

**Affiliations:** 1 Child & Community Wellbeing Unit, Melbourne School of Population and Global Health, University of Melbourne, Melbourne, VIC 3010 Australia; 2 The Royal Children’s Hospital, Melbourne, VIC 3052 Australia; 3 Department of Paediatrics, The University of Melbourne, Melbourne, VIC 3010 Australia; 4 Murdoch Children’s Research Institute, Melbourne, VIC 3052 Australia; 5 Centre for Mental Health, Melbourne School of Population and Global Health, The University of Melbourne, Melbourne, VIC 3010 Australia; 6 Indigenous Health Equity Unit, Centre for Health Equity, Melbourne School of Population and Global Health, University of Melbourne, Melbourne, VIC 3010 Australia

**Keywords:** Disability, Quality of life, Cerebral palsy, Child report

## Abstract

**Background:**

Cerebral palsy (CP) is one of the most common childhood disabilities, impacting many areas of a child’s life. Increasingly, quality of life (QOL) measures are used to capture holistic wellbeing of children with CP. However most validated QOL measures for children are based on adult perspective only, with limited focus on child perspective. Conceptual differences between children’s and adults’ definitions of QOL may reflect different underlying QOL models which contribute to measurement score divergence. This qualitative study investigated the conceptual meaning of QOL for children with CP, comparing child and parent perspectives. Eighteen families completed 8 child interviews and 18 parent interviews. Children (11 boys, 7 girls) represented the spectrum of motor functioning, with comorbidities including epilepsy, intellectual disability, and communication impairments. Child and parent interviews were analysed separately using constructivist grounded theory methods and then findings were integrated to examine similarities and differences.

**Results:**

All participants sought child inclusion in social activities, education, and recreation, requiring negotiation, adaptations, and advocacy. Five conceptual categories emerged from child interviews: socialising, play, negotiating limitations, self-identity, and developing agency. This reflected an individual model of QOL supporting child development goals. Parent interview findings revealed concepts related to child-specific QOL (day-to-day functioning and enabling child goals), as well as parent and family functioning concepts aligned to models of “family QOL”, embracing impacts of family relationships and the interdependence of QOL among family members.

**Conclusions:**

This study identified similarities and differences in child and parent perceptions of QOL for the child with CP. Children provided insights into the importance of play and peer support, and their developing self-identity and sense of agency. Self-directed free play, especially, was identified by children but not parents as a central everyday activity promoting wellbeing and social inclusion. Parents discussed family functioning and aspects outside of child sight, such as managing time and financial resources. Relying on parents’ perspective alone to model child QOL misses valuable information that children contribute. Equally, child report alone misses parent experiences that directly influence child QOL. There is value in incorporating family QOL into parent reports while developing a conceptually separate child self-report QOL instrument.

**Supplementary Information:**

The online version contains supplementary material available at 10.1186/s41687-023-00656-x.

## Background

Cerebral palsy (CP) is one the most common developmental disabilities of childhood, with an approximate prevalence of 1 in every 700 infants (Reid et al. 2016). CP is a lifelong condition impacting many areas of a child’s life beyond the characteristic motor impairments. CP presentation varies greatly, with comorbidities commonly including epilepsy, intellectual disability, chronic pain, and vision, hearing, and communication impairments (Graham et al. 2016). While CP is incurable, interventions aim to improve functioning in activities of daily living and participation in schooling and recreation.

Increasingly, quality of life (QOL) measures are being used in research and clinical practice to capture the holistic wellbeing of children with CP. The World Health Organisation defined QOL as “individuals’ perception of their position in life, in the context of culture and value systems in which they live and in relation to their goals, expectations, standards and concerns” (WHOQoL Group 1995, p. 1405). Based on this definition, QOL can capture the effects of disability on multiple areas of life, and the subjective experience of those effects. It is important to acknowledge that many instruments that measure children’s QOL do not align with the WHO definition of QOL (Fayed et al. 2012).

Established standards for the development of patient-reported outcomes measures such as QOL emphasise the inclusion of target population perspectives in developing measurement conceptual content (e.g. Food and Drug Administration 2009; Reeve et al. 2013), as do clinicians and researchers (Ronen et al., 2022; Willis et al. 2021). While there are now multiple validated QOL measures for children with any disability or chronic disease, and four measures specifically for children with CP (PedsQL-CP, Varni et al. 2006; DISABKIDS-CPM, Baars et al. 2005; CPCHILD, Narayanan et al. 2007; CP QOL-Child, Waters et al. 2006), most are based on parent, clinician and/or expert perspective only, with no or limited focus on child perspective. Even where child input was included in measure development, and where child self-report and parent proxy-report versions exist, they maintain nearly identical format and items (e.g. Baars et al. 2005; Varni et al. 2006). For example, the CP QOL-Child uses nearly identical items for all domains in both versions, with the child version using the item stem “How do you feel about…” while the parent version uses the item stem “How does your child feel about…” (Waters et al. 2006). The parent version has additional domains for family resources, access to services, and parental mental health. Furthermore, a scoping review of QOL instruments revealed that few studies provide detail on the qualitative methods that they use to engage with children to develop instruments (Willis et al. 2021).

Previous quantitative research has found only modest concordance of parent and child scores on QOL measures for children (usually 8 years and older) and adolescents with CP, especially in psycho-emotional domains or those addressing subjective, non-observable topics (Cremeens et al. 2006; White-Koning et al. 2007; Morrow et al. 2012; Davis et al. 2012; Longo et al. 2017). However, there is disagreement about the meaning of parent-child divergence in QOL scores, with some researchers suggesting that parent report is a valid and reliable proxy for child self-report (Varni et al. 2007), or that reported differences may be a statistical artefact (Cremeens et al. 2006).

More than twenty years ago, Eiser (1997) commented on conceptual differences between children’s and adults’ definitions of QOL: these may reflect different underlying QOL models which contribute to score divergence. Qualitative research on how children with CP and their parents respond to QOL scales found children were more likely to rely on single, recent events to determine their responses, whereas parents included events over a longer timeframe (Davis et al. 2007). This may contribute to how children respond to quantitative scales, and their overall perspective of QOL discussed in interviews. Parents generally report lower child QOL scores than their children, which may reflect differences in expectations of what is ‘normal’ or desired for a child with CP if parents use a frame of reference that includes challenges to family functioning and pre-diagnosis expectations of their child’s life. Other researchers have suggested these expectations may contribute to QOL score divergence in combination with differences in response styles (Smith 2011)—both of which may limit the versatility of parent proxy report for their children.

There is limited research on definitions or conceptual models of QOL from perspectives of children with disability generally, and CP specifically, and no disability-specific, child-specific definition of QOL, which is needed to form a child-specific conceptual model of QOL for children with disability. Therefore, this study used a qualitative approach to investigate the conceptual meaning of good QOL for children with CP from the perspectives of children and their parents and compare similarities and differences between these perspectives.

## Methods

### Recruitment

Families were recruited through the Victorian Cerebral Palsy Register (VCPR), an independent, ethically-approved register of individuals with CP. Purposive sampling was used seeking diversity of CP functioning based on overall level of motor function, based on recorded Gross Motor Function Classification System levels (GMFCS; Palisano et al. 1997) ranging from I (most functional) to V (least functional). The VCPR manager emailed 80 families a study description, with a target recruitment of 20 families pragmatically determined on the basis of researcher capacity (Vasileiou et al. 2018) and to allow for purposive sampling. Families responded directly to the VCPR. Details of interested families and non-responders were forwarded to the researchers for follow-up.

Families (including child interviewees) were eligible if the child had a CP diagnosis and could be interviewed in person in Melbourne, or by telephone for parent-only interviews. Previous QOL researchers suggest that younger than 9 years old, children have difficulty completing a self-report questionnaire, and may have very different QOL understandings and priorities compared to older children or adults (Eiser et al. 2000). Therefore, child participants were restricted to 9–12 years. A broader child age range (4–12 years) was applied to parent participants to match most existing QOL tools for children with CP. A child’s ability to participate in an interview was based on parent report and not restricted by the child’s functional abilities (including intellectual abilities).

The University of Melbourne Human Research and Ethics Committee granted ethical approval (project ID 1646922.1), and the VCPR Manager approved the recruitment protocol prior to study commencement.

### Data collection

All interviews were conducted by the first author (ES), who was trained and experienced in conducting research interviews with adults, but with limited experience of disability contexts. Parents of potential participant families were contacted by phone by ES to confirm eligibility, provide information, and arrange the interview. Interview location was determined by family preference, with the participant’s home recommended for child interviews. Formal written consent was obtained at interview, or by prior arrangement for those conducted by phone/Skype.

Interview questions were general in nature, allowing participants to raise specific activities, experiences, or emotions that they experienced in everyday life. Questions primarily focused on concepts of ‘wellbeing’, health and happiness, in keeping with definitions of QOL requiring positive aspects. Prompts also focused specifically on considerations and consequences of the child’s impairments, to elicit data specific to living with CP. Child interviews used a flexible child-friendly approach, including multiple strategies such as visual prompts and drawing materials. Additional file 1: Appendices include further details on child interview strategies and child and adult interview guides.

Interviews were audio recorded and verbatim transcriptions were imported into NVivo 11 for analysis.

### Data analysis

Child and parent interview transcripts were analysed and are reported separately. Drawings created in the course of child interviews were used as prompts for discussion, not as data for analysis. Analysis followed constructivist grounded theory methods (Charmaz 2014), including simultaneous data collection and data analysis, allowing later data collection to be flexibly shaped by early analyses. Notes and memos taken during data collection were used during analysis in addition to interview transcripts. The analysis process was iterative, moving between transcripts, coding, and analytical memos. All interviews were coded by ES, with coding by ED on one interview for comparison and detailed discussion of emerging codes and themes with ED and LG, and more broadly with all co-authors. Analysis was complete when saturation of analytical categories was reached and additional analysis yielded no new interpretations of the data. For additional minor themes and diverse presentations beyond the main categories presented here, see Swift (2019).

For anonymity and confidentiality, all participant names were removed once transcription was complete. However, because of the small number of the children involved and the desire to humanise them and enhance their voice in the research, pseudonyms are used to report child quotes. Pseudonyms were chosen based on relatively common baby names for the years in which the children were born. Parent pseudonyms were not used, as the larger number (19) made this more impractical.

## Results

### Participants

Eighteen families completed 26 interviews—8 child interviews and 18 parent interviews. Of 18 total children, there were 11 boys and 7 girls spanning all GMFCS levels (GMFCS I = 4, II = 4, III = 5, IV = 1, V = 4) with varied comorbidities including epilepsy, intellectual disability, and communication impairments. Three children had fraternal twins without CP. Children of parent only interviewees had a mean age of 9 years and 1 month (range 5–12 years). The child interviewees (2 girls and 6 boys) had a mean age of 10 years (range 9–12 years), and were at GMFCS I (2), II (2), and III (4).

Parent interviews included the child’s primary caregiver (16 mothers, 1 father) only, with one including both parents. Families were located from inner city to outer suburbs within the greater Melbourne metropolitan region, plus one regional town. Additional individual demographic details (such as age, education) were not asked of parents, as the focus of interviews was the child.

Disability service access varied depending on child’s disability severity, family location and financial situation. All families received some government provided disability support. All parents raised issues of cost for services or equipment. As all children were school-aged, during school hours they attended schooling, either public or private mainstream or special schools. Outside of school hours, childcare arrangements varied across families. Some parents received additional caring support after school hours, or accessed occasional respite services. Some families were not eligible for state sponsored caring support or could not pay for support, and care for children was divided between parents. Several mothers did not work in order to provide care for their child.

In 15 families, the child’s parents were partnered and co-habiting; one child’s parents had separately re-partnered and were co-parenting; two mothers were single parents with limited paternal involvement. Two families spoke English as a second language; another family was multilingual. Cultural background and language differences were not raised as significant issues, except that some families were geographically distant from extended family and support systems.

Each participant completed a single interview only. Child interviews lasted between 34 and 71 min (mean = 52.5), and parent interviews lasted between 33 and 75 min (mean = 51.3). Where both parent and child were interviewed, interviews took place consecutively, face-to-face, at the family home. Parents chose to be present for three child interviews; for five child participants parents were absent for most or all of the child interview. Parent only interviews occurred in the family home (6), at a children’s hospital (2), by phone (1), or by Skype (1). Interviews occurred from January to June 2017.

### Child interview findings

Analysis of child interviews revealed five main conceptual categories: ‘social beings; being social’, ‘play’, ‘negotiating limitations’, ‘defining the self’, and ‘establishing agency’, described below. All five categories were related and were underpinned by social relationships. Categories were based on active processes that functioned as developmental goals for the children, varying by age of the child respondent and changing environmental demands. The disability-environment interactions added additional facets such as restricted opportunities due to developmental challenges, unlikely to be faced by typically developing children.

#### Social beings; being social

Child interviewees discussed both the importance and enjoyment of spending time with others, as well as the processes involved in being social (such as how to make friends, or maintain friendships), and situations and people that provided help or hindrance to their socialising. This was often first raised in the context of peer friendships, as Ana said, “I like hanging out with my friends”, or Nicholas stated he was like the happy emoji “when I’m playing with my friends”.

Children also expressed enjoyment and appreciation of relationships with their parents and siblings. They mentioned extended social networks in which they singled out valued relationships with other adults, including extended family, carers, teachers, school aides, therapists, and other community members they engaged with in leisure activities. Among the common experience of disability-related exclusion, sympathetic adults who facilitated opportunities for inclusion were highly valued by all the children (see ‘negotiating limitations’ for examples).

#### Play

Play was a central and highly valued activity in children’s lives. Children played with siblings, friends, pets, games, and toys; play involved “running around” outside, sitting down indoors, or some combination. For older children, ‘play’ transitioned into ‘doing stuff’ with friends. Schoolwork or adult directed hobbies were not play.

Disability-adapted or alternative equipment could be scarce, unaffordable, or hard to access, resulting in children being excluded from play. A few of the children faced additional limitations through rules and regulations in mainstream environments. Eric was not allowed in his wheelchair on the grassed area of the school playground during recess, “where all my friends are”, unless pushed there by his aide or another adult. When adults were unavailable, he missed out on play. Exclusions from play impacted children’s opportunities for peer socialising, and exclusions from social opportunities limited possibilities for play. As Sofia said, “I wish I had friends in this street so I can play”.

#### Negotiating limitations

Children reported negotiating between personal abilities and impairments, environmental limitations, and availability of assistance, alternatives, and adaptations. The physical limitations of CP could be outweighed by co-occurring conditions such as intellectual disability, anxiety symptoms, or recurrent illness. Children were self-aware of their limitations and resulting exclusions. Listing the multiple physical hobbies of her siblings, Sofia said, “there’s not that much stuff to do for me”. Limiting environmental factors included physical (e.g. school areas inaccessible for mobility aids), and non-physical barriers such as attitudes of authority figures or rules and regulations (e.g. a school preventing a child who uses a wheelchair from participating in the wheelchair-friendly game skittles after he was once hit by the ball).

Help could overcome barriers when it was identifiable and accessible. Zachary said that at his new school he liked “The nice teachers. [Because] Like, if you’re in trouble, they’ll help you out and stuff”. Sofia went further in directly contrasting identifiable help at her new school where “if you’re feeling angry you go to that person”, and her previous school saying, “but at that school there was no one”. Some children also mentioned regularly receiving help from peers and siblings, showing that inclusion could result directly from child social networks and be negotiated without adult oversight.

#### Defining the self

The tension between ‘normal’ and ‘different’ framed children’s developing self-identity and self-esteem. As Zachary said: “I’m still normal and everything. It still feels like I’m a bit different.”

Externally, children’s self-identity is developed and defined by the duality between visible difference and the desire to be included as ‘normal’ and treated similarly to and by others. Sometimes visibility could be useful—wheelchair-user Gus was referred to as “the golden child” within his family; his disability enabled them special access that they would not otherwise receive.

Internally, children’s sense of self and self-esteem developed in the context of self-awareness in disability-related differences in abilities. Peer comparisons could jeopardise self-esteem when children were habitually unable to match typically developing peers. Supportive, encouraging relationships helped children to make positive self-appraisals about themselves and their disability. Additionally, disability-specific alternatives and adaptations enabling opportunities to achieve were invaluable. Caleb discussed sports at his special school, “We jump over hurdles and there was basketball. I got to win!”—while he enjoyed the sports, he expressed the most animation about winning, something that was not possible at his previous mainstream school.

#### Establishing agency

Agency has been defined as “to intentionally produce certain effects by one’s actions” (Bandura 2017, p.130). Agency is not synonymous with autonomy or independence, especially for children physically dependent on others for basic needs. For children with CP, agency may be eroded by diminished physical control over their own bodies, from CP impairments, and from medical interventions. In the healthcare context, children raised issues of discomfort resulting physically from medical interventions, as well as their perceived lack of control in relation to being excluded from decisions about their bodies and functional priority setting. Zachary was able to enact his agency in a long-term supportive physiotherapy relationship, saying “if I’m not comfortable with doing something I will say, like, “I’m not really sure about doing that. Can we try something else?”,” but reflected that a first-time physiotherapist visit could be quite different as “you could be really nervous and you just don’t know how it’s going to go…”.

Social relationships in the family and with other trusted adults facilitated child agency, through support and encouragement of children to explore autonomous actions and choices. Others’ expectations had consequences for child agency. While Ana said, “Just people seem like they underestimate me” with a sense of frustration, Nicholas exploited low expectations in the classroom, saying “Normally I let my aide do all my work”. In both cases, excessive intervention limited children’s opportunities to exercise agency and instead encouraged dependencies, despite Nicholas’ enjoyment of doing less schoolwork.

### Parent interview findings

Parent accounts of their child’s experiences were not chronological. However, analytical themes presented a narrative that broadly followed the timeline of their child’s life, juxtaposed with past parental experiences with their child’s diagnosis of CP and the subsequent navigation of medical and disability services. Parents often included experiences from their child’s infancy that the child was unlikely to recall. The categories overall covered two areas: (1) parent and family QOL, focussing on parental experiences and wider family concerns, and (2) child specific QOL, including children’s present day-to-day needs and future considerations.

#### Parent and family QOL

Though parents were asked to describe their child in present tense, the arc of the parent narrative began with parents’ past experiences of the process of diagnosis, and ‘assimilating’ this knowledge into their expectations of their child and their child’s life. Subsequently, one significant aspect of parents’ ongoing perspectives of their child’s disability was how parents found positivity in a situation that was often distressing and challenging. For example, one parent concluded that, in spite of her daughter’s significant physical and communication impairments, “she still is quite happy and she’s got a lot of people around her that love her and support her” (parent #17, child 11 years old, GMFCS V). Several others reflected that other families had a more difficult situation. Lastly, parents emphasised the love they had for their child, inclusive of their CP. One parent raised this as an active part of parenting a child with disability:

The world can give you all sorts of grief but if you’ve got a comfortable and lovely home to come home to with lots of love, then that becomes less significant. (parent #9, child 5 years old, GMFCS I).

Parent and family QOL categories in the present encompassed the competing demands of parent needs, needs of other family members, and the time and effort required to provide good care to the child with CP. Competing demands created practical difficulties in managing day-to-day family life, which often involved making trade-offs rather than managing everything. This required a balance between not only necessary tasks, but the cost of each additional opportunity for the child with CP or other family members.

Future perspectives of parental QOL related to two ongoing challenges. Firstly, many parents experienced challenges to mental health that are well documented in previous literature (e.g. Bourke-Taylor et al. 2012; Emerson and Llewellyn 2008; Gilson et al. 2018), such as having to give up employment to care for their child, additional financial pressures of disability-related costs and missing out on social or recreational opportunities.

Secondly, parents lived with ongoing, everyday uncertainty. This could result directly from their child’s disability, from recurrent illness or pain, the longer-term uncertainty of how the child’s symptoms might change with ageing, or uncertainty in funding of necessary services, or the lack of choice of when or where a child could access specialist medical care. As a result, all plans for the child, the family, or the parent themselves, were tenuous. For example, one parent explained, “It’s ‘okay, what’s in the immediate future?’ and you, sort of, have a vague underlying long-term plan” (family #6, father, child 12 years old, GMFCS III). The need for a sense of future support plans caused tension with parents’ inability to realistically plan for the long-term future, as one mother said, “we don’t know what the world will look like. We don’t know what his needs will be” (family #6, mother, child 12 years old, GMFCS III). This created an additional barrier to accessibility for parents’ own mental health and a multi-layered interplay between parental mental health and child QOL outcomes.

#### Child-specific QOL

Within parent interviews, analysis of parents’ reports of child-specific QOL revealed hierarchical categories, from immediate needs (everyday care), to short-term goals (enabling accessibility and inclusion), to the broader disability system level (addressing system gaps and future needs).

Everyday disability care involved day-to-day demands, project management, and interceding in therapeutic relationships. Day-to-day demands of disability-specific care have been previously recognised as additional work that impacts parents (e.g. Murphy et al. 2006; Raina et al. 2005). Parents here reported additional work that is less frequently cited, including balancing disability care and other aspects of family functioning as a type of project management. Parents were necessarily responsible for managing therapeutic relationships between themselves, their child, often multiple health professionals and support workers, and making decisions about interventions. One single mother recalled medical decision making for her daughter, saying starkly, “It would literally just come down to me … to do everything I can to help her fight and whatever it needs” (parent #13, child 7 years old, GMFCS II).

Once everyday needs were managed, child goals centred on inclusion, education, social life, participation in recreation, and the opportunity to take family holidays. These were the personal goals parents held for children to fulfil their dreams and be happy. Parents exerted significant effort in finding and choosing opportunities for their child in each of these areas to allow them to achieve their dreams, to participate and be included—in whatever way this was meaningful for the child and their family. This parent encapsulated those goals, while discussing her daughter learning to ski:

She may not be able to do it, but we wanted to encourage her, we wanted her to try, we wanted her to have that sense of freedom, of moving unencumbered. (parent #5, child 11 years old, GMFCS II).

Every parent could quickly identify system gaps and unmet needs missing from their ideal environment for their children and had suggestions to improve access and services. As a result, parents became advocates for their child. For example, one parent of a non-verbal child with Level V CP explained, “My son can’t speak out … I just be his voice. We speak out for him” (parent #14, child 8 years old, GMFCS V). Parents expended significant energy in advocacy activities to enable their child to achieve their recreational or social inclusion goals, such as fighting for equitable inclusion in services and activities and access to physical spaces.

## Discussion

The QOL issues raised by both children with CP and their parents in this study reflected multidimensional definitions of QOL, including functional outcomes, personal achievements, and inclusion in education, social life, recreation, and family activities. While many of the goals for the child’s QOL were shared by children and parents, their perspectives often differed. Both groups noted that achieving goals relied on external supports for participation and inclusion. Parents also provided insights into their role in managing disability care and the competing family demands.

### Unique child perspectives

Children provided a unique perspective on their developing self-identity as independent and social beings in the different contexts of the home, school, and community, and how this was influenced by their disability. Children also provided insight on complex issues of social inclusion specific to CP, from negative experiences of bullying and exclusion to positive experiences with disability specific services, and adaptations during school and community activities. These findings echo research developing a multi-domain framework of self-concept for children with CP (Cheong et al. 2016).

Often these perspectives reflected child experiences where their parent was not present – for example, play time at school, or recreational time with peers. This highlighted the value of peer support for children with disabilities, as has been noted by previous researchers in the school context (De Schauwer et al. 2009). Parents reflected on adult support children received but did not generally discuss support children received from peers. Peer support contributed to positive self-concept for children through building social relationships and promoting inclusion with a level of independence beyond activities requiring adult support.

While children expressed fewer practical concerns than parents in accessing treatment, they raised the need to be included in treatment goals and plans. These rights are codified in the Convention of the Rights of the Child (United Nations 1989). Establishing an increasing sense of independence and agency is a significant developmental process for all children and adolescents (Patton et al. 2016). Previous researchers have described lower levels of engagement and greater passivity for young children with disabilities in a setting in which adult actions were more directive with limited opportunity for child individuality in response (Nind et al. 2010).

Play was not recognised by parents as valuable to children’s QOL outside of participation and inclusion goals, whereas it was a central everyday activity for children and promoted wellbeing as well as social relationships and inclusion. For all children, free play provides opportunities for learning and contributes to child development (Missiuna and Pollock 1991). Definitions of free play from research literature suggest, among other things, that it is: universal and central (Factor 2009); the ‘primary productive activity of childhood’, fun, and self-motivated (Missiuna and Pollock 1991); not ‘serious’ or ‘real’ and must involve freedom of choice for the child (Mindes 2015). Factor (2009) recounted exchanges with children about play that were very similar to these findings.

### Unique parent perspectives

Parent perspectives not shared by children related mainly to either parents’ own or broader family functioning. Overall, children did not discuss these factors, however, they impact on child QOL, both through family QOL mechanisms as well as practical considerations. Especially in the clinical context, information about parental issues can shed light on child QOL, and impact how interventions in the family can be implemented for the best outcomes. Including parental concerns in clinical goals is necessary for parents to implement child-specific interventions for children—as documented in family-centred approaches (Dickens et al. 2011).

Living with uncertainty was an ongoing concern for parents but was not raised by children. This may have resulted from two factors. Firstly, adults are generally much more likely to consider the future than children. This difference in focus has been shown in the discordance between child and adult responses to child QOL questionnaires, with child responses generally based on a significantly shorter timeframe (Davis et al. 2007). Secondly, in Australia most children are diagnosed with CP during infancy (Novak et al. 2017); as such, they have no memory of not having this diagnosis. In contrast, most of the parents interviewed described in detail the period of time leading to their child’s CP diagnosis—usually beginning with an assumption of a healthy pregnancy or infant without CP. For parents, the narrative of their child’s CP therefore begins with change, whereas children experience CP diagnosis as a stable fact.

### Similarities and differences in perspective

Some differences discussed here may be related to differences in CP functioning. Children interviewed were at GMFCS I, II, III, with sufficient communication and intellectual ability to complete an interview with limited assistance, whereas parent interviews included parents of younger children and those with more severe CP. Some CP-related uncertainty parents experienced, such as comorbidities and frequent illnesses, increase with greater CP complexity. However, overall, child’s GMFCS level of CP was not related to parent interview findings. This suggests that child and parent findings reflect different child and parent perspectives rather than differences in CP functioning.

There is limited qualitative literature comparing child and parent experiences of disability, however other researchers have found similar perspectives. Garth and Aroni (2003) showed that although both parents and children reported the importance of communication with healthcare professionals, their experiences and priorities for healthcare communication differed. Similarly, in our study both children with CP and their parents wished for supportive environments, social networks, inclusion and participation, but how they perceived these differed. This mirrors other qualitative research on wellbeing with typically developing children (Sixsmith et al. 2007) and children with CP (Parkinson et al. 2011). Both these studies found overall agreement in many areas of life but differences in details, such as recognition of the value of pets or alone time in children’s social lives.

One unanticipated finding was that considering both family QOL and individual QOL may be the best way to improve conceptualisation of QOL and wellbeing for children with CP, and to target interventions aimed at improving QOL. Family QOL recognises the interplay between the individual QOL of family members, their interactions, and collective family functioning (Boelsma et al. 2017). This reflects the findings of the parent interviews and could be incorporated into parent reports. For example, measures of family QOL can identify barriers to the pursuit of family goals, such as the need for additional supports to enable improved family functioning (Brown et al. 2006). A study comparing functioning of families with two different neurodevelopmental disabilities found parallels in the impact of family and contextual factors on QOL (Brown et al. 2006). This suggests that a family QOL perspective may also generalise to families of children with disabilities other than CP. Previous researchers have conceptualised family QOL models to incorporate both individual QOL of separate family members and whole of family concerns, and interactions between them (Poston et al. 2003). One drawback of the family QOL model in disability research is that measures have focused mainly on negative impacts of disability on the family, as in the PedsQL Family Impact Module (Varni et al. 2004).

### Limitations

The data reported here included families in and around Melbourne with a child with CP between 4 and 12 years. While there were variations in experiences within the sample, these results may not generalise outside of this geographic area, and it should be noted that in all but one family the parent interviewee and primary caregiver was the child’s mother. Additionally, only a small number of children were interviewed directly. Though this allowed for focused in-depth analysis of their experiences, future research could continue to explore the concepts from this study with other groups of children with CP, and potentially from fathers as well. Other future researchers seeking to understand QOL for children with CP should use multiple methods to understand experiences of children themselves, as well as parent perspectives, and the wider context of family interactions. In practical terms for clinicians, this may mean using a proxy report to understand parent views of child QOL, then conducting a conversation with child and parent separately to understand child perspective and family dynamics, respectively.

## Conclusions

This study showed clear commonalities and differences between child and parent perspectives of QOL. Both parents and children provided a multidimensional understanding of QOL beyond functional constraints of CP, and both groups sought child inclusion in recreation, education, and social activities that required negotiation, adaptations and advocacy. However, children provided additional insights into their developing self-identity, sense of agency and the importance of play and peer support. Self-directed free play, especially, was identified by children but not parents as a central everyday activity promoting wellbeing and social inclusion. Parents discussed family functioning and aspects outside of child sight, such as managing time and financial resources. These differences have implications for QOL measurement. Relying on parents’ perspective alone is likely to miss valuable information that children can contribute. Equally, child report alone would miss many aspects of parent experience directly influencing child QOL. There could be value in incorporating family QOL into parent reports while developing a conceptually different child self-report QOL instrument.

### Supplementary Information

**Additional file 1.** Appendices.

## Data Availability

Data created and analysed in this study are not publicly available in accordance with the ethical approval, due to potential for re-identification and breach of confidentiality.

## List of abbreviations

CP: Cerebral Palsy
QOL: Quality of Life
GMFCS: Gross Motor Function Classification System
