# Supplementary material for: Qualitative differences in perspective on children’s quality of life between children with cerebral palsy and their parents
Source: J Patient Rep Outcomes. 2023 Nov 20;7:118. doi: 10.1186/s41687-023-00656-x (PMC10661547; doi:10.1186/s41687-023-00656-x)
Supplement: Supplementary file 1 — Additional file 1. Appendices. [file 41687_2023_656_MOESM1_ESM.docx]

# Appendix 1: Child-friendly interview methods and child interview guide

## Description of child-friendly interview methods (extract from Swift, 2019)

Several strategies were planned to engage child participants. These included 1) seeking parent collaboration prior to the interview to gauge a child’s abilities and interests; 2) a warm-up activity to begin the interview, and 3) multiple activity props used throughout the interview including drawing materials, and various printed pictures and prompt cards. A combination of these was used in each interview, with planned strategies for each interview varying based on the child’s age and the parent’s opinions of their abilities. Throughout data collection, these strategies, prompts, and scripts were refined and adapted based on how children in earlier interviews engaged with them or commented on them.

*Warm-up activity*

A warm-up game was prepared to begin each child interview, to help the researcher to gauge the child’s interests and abilities and increase the participant’s comfort and reduce their anxiety. This was based on the interviewer card-game warm-up activity described by Teachman and Gibson (2013), which their child participants found engaging. The game is based on two identical sets of cards pre-printed with questions, with one set provided each for the researcher and child participant. The child and researcher then take turns to read and answer the questions on the cards. The child is encouraged to play interviewer first by choosing a card to ask the researcher. The questions used here were: “Can you tell me a little about yourself?”, “What is something you’re really good at?”, “If you could have any super power, what super power would you choose?”, and “If you were to be any animal in the world, what animal would you be?”. This activity was introduced to each child as a game for the child to get to know the researcher a little before the interview, and because it would be unfair for only the researcher to ask questions.

*Interview activities*

Several activity options were introduced throughout the main part of the child interview to make the questions more interesting and fun. Prepared activity props included 1) colour printed emoji faces, 2) pens, pencils, and large paper for drawing and 3) written prompt cards for use in drawing activities. All printed materials were affixed to stiff cardboard to make them easier to handle. Most of the materials were visible from the beginning of the interview, so that if the child expressed interest in specific activities, these would be used first. Otherwise, the researcher directed the child to activities that might be suited to their interests or abilities based on information from the parent. All materials involving written text were intended to be read aloud by the interviewer as they were used; in a few cases parents assisted children in reading materials aloud.

### Emoji

The emoji faces were used initially as a representation of how the child has been feeling lately “Which one of these is most like you? … Why did you choose that one?”. These could also be combined with other drawings or discussions as prompts for eliciting the child’s feelings or applied to specific areas of a child’s life such as school, family, or other activities.

### Drawing materials

Drawing materials were provided primarily as a distraction during the interview rather than for visual data collection. However, they were also used to prompt conversation, for example for children to draw things they mentioned or things they enjoyed. The interviewer was then able to ask questions about the drawings and their meanings.

*Prompt cards*

Printed prompt cards could be used as prompts for verbal discussion or incorporated into other drawing activities. There were four prompt cards, with text including “places I go”, “things I do”, “people I meet”, and “feelings I have”. These phrases were chosen based on their successful use in previous similar QOL research (Parkinson et al., 2011), to cover a range of issues relevant to children’s experiences of QOL. As they are more directive than some of the other methods, they were intended to be used where the child appeared to have difficulty responding to other more open activities or questions, to begin discussions about these areas of life.

*Analysis of engagement strategies*

A secondary aim of the analysis was to critique the interview methods used with children. This involved reflecting on multiple levels within the interviews. Firstly, there were practical considerations of applying the chosen strategies with the children—such as the ease of use, children’s interest or lack of interest, and any other similar practical consequences. Secondly, there were specific considerations of the influence of prompted activities on the content of interviews with the children—such as whether the use of emoji or prompted drawings elicited rich or detailed discussion of QOL. Lastly, there were broader reflections on the interactional process and how these strategies may have influenced the work of the interviewer and participant within the interview. For example, Rapley’s review of qualitative interview research (Rapley, 2012) exposes the interactional nature of qualitative interviews, including the active work of the interviewer in guiding participants and the position of participants in creating the narrative and their own identity. His recommendations for explicitly considering the interactional process in the analysis fits easily within the constructivist perspective, and are echoed by Potter and Hepburn (J. Potter & Hepburn, 2012). Furthermore, the recommendations of these authors together provide a way to evaluate how the specific strategies may have contributed to data produced in the child interviews. Therefore, especially in relation to the use of child-friendly materials, this level of analysis considered 1) how the interviewer’s questioning and prompting guided the children’s responses and way of responding, 2) contextual assumptions and identities children created within the interviews, 3) the roles of interviewer and child within the interview with respect to power and the co-construction of the interview content. The full results of this reflexive critique of child interview methods and influence on interview content are available in Swift (2019).

## Interview Guide and example questions

The interview guides provided initial prompts for interviewees, but interviews were open-ended and flexible, so that not all questions were asked of all participants if the main areas the interview guide addressed were introduced by participants. After initial questions taken from the interview guide, interviews were led by topics introduced by participants themselves, as well as later interviews including specific issues and topics raised by earlier participants.

Questions for children, especially early in each interview, focused predominantly on positive aspects of their lives, such as their favourite pastimes, friends, or enjoyable aspects of schooling and hobbies. However, when children introduced negative experiences, these were also explored with additional prompts. Once children appeared to be comfortable in the interview discussions, they were also asked if they had other negative or uncomfortable experiences related to having CP.

At the end of each interview, both parents and children were asked if they would feel comfortable discussing the QOL issues raised in the interviews, with their health care or disability service providers. For example, if they felt it would be appropriate or useful for their health service to ask them to complete the CP QOL-Child or a similar measure. These questions were intended to gauge whether families felt QOL issues were relevant to the services they currently receive.

*Interview Guide*

I would like to know about different parts of your life, like your family, your friends, your health, or your school. Tell me what are the things you enjoy?

- What is your favourite thing about ____________?

Tell me about the things you find challenging in your life.

[showing child series of different emoji faces] which one of these do you think is most like you lately? what made you choose that one?

- Further prompts depending on response – e.g. what made you feel like that?

For children capable of self-reporting on the CP QOL-Child questionnaire: Do you think you would be comfortable to answer a questionnaire about these sorts of things, like your feelings about your life, if your physio/doctor/[other health service child has mentioned] asked you to?

## References

Parkinson KN, Rice H, Young B (2011) Incorporating Children’s and Their Parents’ Perspectives into Condition-Specific Quality-of-Life Instruments for Children with Cerebral Palsy: A Qualitative Study. Value in Health 14:705–711. https://doi.org/10.1016/j.jval.2010.12.003

Potter, J., & Hepburn, A. (2012). Eight Challenges for Interview Researchers. In: Gubrium J, Holstein J, Marvasti A, McKinney K (eds) The SAGE Handbook of Interview Research: The Complexity of the Craft. SAGE Publications, Inc. https://doi.org/10.4135/9781452218403.n39

Rapley T (2012) The (Extra)Ordinary Practices of Qualitative Interviewing. In: Gubrium J, Holstein J, Marvasti A, McKinney K (eds) The SAGE Handbook of Interview Research: The Complexity of the Craft. SAGE Publications, Inc. https://doi.org/10.4135/9781452218403.n38

Swift EC (2019) What is quality of life for children with cerebral palsy? A constructivist grounded theory approach with implications for measurement. Dissertation, University of Melbourne.

Teachman G, Gibson BE (2013) Children and youth with disabilities: Innovative methods for single qualitative interviews. Qualitative Health Research 23:264–274. https://doi.org/10.1177/1049732312468063

# Appendix 2: Parent Interview Guide

- Tell me about your child’s life / your child – how they spend their time, what things they enjoy?
- What do you think are the most important things for your child to be well and to have a good life?
  - Prompts if not covered by initial answers: e.g. at home, at school, in the community, in terms of health, in the future…
  - possible additional prompts: CP specific questions – eg is this different because of your child’s impairments?, if they don’t raise limitations – are there things that prevent your child having a good life?
- We are planning to use this research project to create a new survey form for health professionals to learn about the QOL of children with CP who they are treating. How do you feel about your child’s health professionals asking you and them about your child’s QOL?
  - Are there things you would not want to discuss with your child’s health professionals? prompt – such as family finance or difficulty accessing other services, or your own health and QOL
